# A new treatment approach to conduct disorder and callous-unemotional traits: an assessment of the acceptability, appropriateness, and feasibility of Impact VR

**DOI:** 10.3389/fpsyt.2025.1484938

**Published:** 2025-05-01

**Authors:** Nicholas D. Thomson, Salpi S. Kevorkian, Laura Hazlett, Robert Perera, Scott Vrana

**Affiliations:** ^1^ Arche VR LLC, Glen Allen, VA, United States; ^2^ Department of Surgery, Virginia Commonwealth University, Richmond, VA, United States; ^3^ Department of Psychology, Virginia Commonwealth University, Richmond, VA, United States; ^4^ Department of Criminology and Criminal Justice, Florida International University, Miami, FL, United States; ^5^ Department of Biostatistics, Virginia Commonwealth University, Richmond, VA, United States

**Keywords:** virtual reality, conduct disorder, emotions, treatment, callous unemotional traits, mental health interventions

## Abstract

**Introduction:**

Conduct disorder (CD) is highly prevalent among youth, yet existing and conventional treatment approaches are limited and costly. Further, most interventions for CD focus on behavior management rather than targeting the underlying mechanisms of CD. To meet the needs of youth with CD (ages 10-17), we developed Impact VR, a virtual reality intervention that promotes competency in emotion recognition and regulation, as well as modeling prosocial behaviors. Impact VR provides immersive storylines and gamification of psychoeducation training. The present study aimed to understand the perceptions of Impact VR for acceptability, feasibility, and usability across key stakeholder groups, including youth with CD, caregivers of youth with CD, mental health practitioners, and educators.

**Methods:**

A total of 60 adults, including mental health professionals (*n*=20), teachers (*n*=20), caregivers (*n*=20), and 20 youth with CD completed a trial of Impact VR and completed surveys.

**Results:**

Results demonstrated a high overall acceptability of Impact VR (95% - 100%) across all groups, and a high approval rating for intervention appropriateness (98.75% - 100%) and feasibility of the intervention (97.50% - 100%) across stakeholder groups. The majority of youth (90%-100%) reported that the skills learned would improve their mental health and relationships with friends, parents, and teachers.

**Discussion:**

Findings revealed that youth with CD and key stakeholder groups rate Impact VR favorably and positively. Future research is needed on the effectiveness of Impact VR in improving emotion recognition and reducing CD symptoms.

## 1 Introduction

Conduct disorder (CD) is one of the most prevalent childhood psychiatric disorders, and epidemiological studies estimate that CD is responsible for 5.75 million years of healthy life lost, with a global burden in children surpassing common mental disorders (1). CD is diagnosed based on the presence of aggressive behavior toward people and animals (i.e., physically cruel to people), destruction of property, deceitfulness or theft (i.e., “cons” others), and serious violation of rules that cause significant impairment in social, academic, or occupational functioning (2). Within the diagnosis of CD, a specifier of Limited Prosocial Emotions (also termed callous-unemotional [CU] traits) can be made if the youth displays a persistent lack of remorse/guilt (i.e., not remorseful after hurting someone), a callous lack of empathy (i.e., cold and uncaring), unconcerned about performance, and shallow or deficient affect (i.e., displays shallow, insincere, or superficial emotions). Between 12% to 46% of youth with conduct problems present with significant levels of CU traits (3–7). The CU traits specifier designates a subgroup of youth who are at greatest risk of criminal and disruptive behavior, leading to long-term antisocial behavior and mental health problems seen into adulthood (8–10). CU traits can be further categorized as either primary or secondary based on the presence or absence of anxiety (11). Similar to Karpman’s (12) subtyping of psychopathy in adults, primary CU traits are related to low anxiety, fearlessness, and deficits in emotion processing (13, 14). In contrast, secondary CU traits are associated with higher levels of internalizing symptoms (e.g., anxiety and depression), hyperactivity to threats, impulsivity, and emotion dysregulation (14). Secondary CU is believed to arise from adverse or abusive home environments, whereas primary CU is thought to be shaped largely by biological risk factors (14, 15). As a result, it has been argued that these subtypes are important for directing more tailored treatment planning, as the subtypes have very different treatment needs (16). For example, youth with primary CU traits may benefit from improving social skills, affective empathy, and emotion recognition deficits (17). Secondary CU traits may benefit from strategies that address emotion dysregulation and impulsivity (18, 19). Given that there is variability in contributing factors among youth with CD and CU, treatment approaches that target core mechanisms of CU and CU subtypes may be most beneficial (e.g., emotion recognition, emotion regulation, social skills training). Unfortunately, existing treatment approaches for adolescents with CD are limited, and those that demonstrate promise largely focus on behavior management rather than addressing the underlying mechanisms of CD and CU traits (20).

Current approaches to treating CD and CU traits mainly focus on reducing antisocial behaviors rather than delving into the root causes of the disorder, which may limit the potential for lasting improvement (20). The most effective strategies available for youth with CD and CU traits provide around-the-clock case management for both the affected youths and their families, such as multisystemic therapy (MST; see 21 for meta-analysis detailing effects of MST), alongside offering comprehensive parenting guidance to navigate challenging behaviors, similar to other common, evidence-based family treatments (e.g., Functional Family Therapy; see 22). However, these interventions may fall short of addressing key barriers faced by youth with CD and CU traits. For instance, youth with serious antisocial behavior often have language deficits (23, 24), such as difficulties in processing language and abstract concepts, which can hinder engagement with verbally intensive therapies, limiting the effectiveness of traditional approaches. Additionally, motivational challenges, such as reduced sensitivity to social rewards and diminished emotional engagement, make it difficult to sustain participation and adherence among this population (9, 20). While these family-based treatments have been effective in reducing criminal behavior and violence among youths identified as delinquent or exhibiting CU traits, they have not specifically been shown to diminish the symptoms of CU traits. Additionally, these intensive treatment options come with a hefty price tag and demand considerable resources from communities and schools to maintain a continuous mental health support system throughout the year. Studies indicate the cost of implementing such intensive treatments for youth with disruptive behavior problems ranges from $5,000 to $10,000 per child. Despite extant findings detailing potential returns on these therapies, the long-term financial cost savings, while seemingly promising, necessitates a substantial upfront investment from schools and hospitals (25), without guaranteed evidence of mitigating CD and CU symptoms directly. Therefore, there is a need for effective new treatment approaches that require less upfront cost.

A novel approach to treating CD and CU traits is to focus on addressing transdiagnostic processes, particularly emotion recognition deficits, which are common across various childhood disorders (26). Research has shown that youth with CD and CU traits, similar to those diagnosed with disruptive behavior problems (e.g., oppositional defiant disorder) and neurodevelopmental disorders (e.g., attention-deficit/hyperactivity disorder [ADHD], autism spectrum disorder), have deficits in recognizing and regulating emotions (27). For youth with CU traits, this impairment is especially pronounced with negative emotions such as fear (28) and sadness (29). These deficits in emotion recognition are believed to disrupt social development during childhood. Given the role of emotion recognition in social development, research has explored the role of emotion recognition as a prevention strategy for violence and antisocial behavior. For example, brief emotion recognition training has been found to reduce behavioral problems among children (26), increase affective empathy, and reduce conduct problems among youth with high levels of CU traits (30). Emotion recognition training has even been found to reduce violent crime 6 months post-intervention among high-risk juvenile offenders (31). This suggests that targeting transdiagnostic processes may be a more target and resource-effective approach to addressing CD and CU traits.

Youth with disruptive behavior are notoriously difficult to engage in intervention, especially those most at risk of violence (32, 33). Therefore, a new approach to intervention that engages youth is a high priority. An emerging body of research highlights the benefits of using cutting-edge, immersive virtual reality (VR) technology to deploy interventions that target transdiagnostic processes and factors more broadly (see 34) and highlights the utility of VR-based treatment interventions for emotion regulation more specifically (35). This is particularly salient as prior research has demonstrated the comparability of emotion elicitation in VR to emotion elicitation in real-life scenarios (36), underscoring the potential for high ecological validity within VR interventions (37). Additionally, research using eye-tracking technology found support for conducting emotion recognition training within VR, noting a high degree of similarity in recognition patterns between virtual and real faces (38). This high degree of comparability in virtual to natural and digitized expressions, coupled with the advantageous nature of VR in enhancing elicited emotions and emotional experiences (see 39), suggests that VR may be an effective modality as a treatment approach for CD and CU traits. Furthermore, VR can be used to advance and augment traditional interventions. For example, a case study by Heikkilä et al. (40) implemented compassion-focused therapy (CFT) alongside VR exposure therapy. This program, named CFT+VR, incorporated structured VR sessions featuring exposure to threatening situations, soothing scenarios, and optional interactive activities to enhance emotion regulation, self-awareness, and the embodiment of emotions. The case study, profiling a high-risk youth offender, demonstrated promising results for increasing emotional insights. While there is general support for VR mental health interventions for youth (33), research on adults has demonstrated that they do not have lasting effects (41) or immediate improvements post-intervention for aggressive behavior (42). Therefore, VR interventions may be better suited for early prevention efforts among youth, where the potential for skill acquisition and behavioral change is greater than in forensic adult populations.

The need for VR treatment interventions in youth has been documented as critical given the wide range of applicability of this technology and its potential to enhance treatment efforts given the gamified nature of its design (see 43). Studies conducted before the significant advances in VR technology have provided support for the validity of artificial, or virtual, emotional expressions (44), highlighting the viability of a gamified VR emotion recognition program for youth that can enhance emotion-based learning outcomes through immersive gameplay and storylines (e.g., use of avatars, culturally relevant characters, animation, etc.). Studies of youth in early to middle adolescence with other forms of attention and emotion deficits (e.g., ADHD) have identified better performance metrics and greater levels of preference for VR-administered tasks as compared to computerized tasks (45), underscoring the suitability of these types of interventions for this target population. There is, however, a lack of emotion recognition training programs that are set in real-life settings and contexts (see 43). In response to this need and to address these limitations in the intervention space, a self-guided training program called “Impact VR” was developed for youth (10-17 years of age) to target the mechanisms of CD and CU traits and to teach youth how to effectively identify emotional expressions in others.

Impact VR is an innovative new intervention specifically designed to address core deficits associated with CD and CU traits, including emotion recognition, emotion regulation, and prosocial behaviors. Leveraging the immersive capabilities of VR technology, the program places youth in engaging, socially relevant scenarios where they practice identifying and responding to emotional expressions, navigating complex social interactions, and employing prosocial strategies to resolve conflicts. Impact VR aims to provide an engaging and scalable alternative to traditional interventions. New technologies like Impact VR may hold promise for engaging and treating youth with CD and CU traits; however, early-stage stakeholder input is crucial to ensure the intervention aligns with user needs and preferences. Effectiveness is only impactful if users are willing to adopt and engage with the program. Gathering stakeholder feedback on the acceptability, appropriateness, and feasibility of Impact VR is an essential first step in its development, enabling refinement and optimization for real-world implementation.

Understanding stakeholder perceptions of new interventions is a critical step in the development process, particularly for youth populations who are notoriously difficult to engage in treatment. Over the past decade, stakeholder engagement has become increasingly prevalent and is widely regarded as best practice in health research (46, 47). Many international research funders now require stakeholder engagement as a standard component of research design (48). While patient engagement in adult mental health research is well-established, progress in youth patient engagement appears to be lagging (49). The growing emphasis on stakeholder engagement is driven by the belief that meaningful engagement enhances the quality, relevance, and acceptability of research for all stakeholders involved (50). Indeed, perception data provides valuable insight into the acceptability, feasibility, and appropriateness of an intervention, which are essential for ensuring successful implementation, long-term sustainability, and scalability. Research has shown that interventions that align with the needs and preferences of their target population are more likely to be adopted, yielding higher engagement and better outcomes (51). For youth with CD, who often face systemic barriers to care and resist conventional interventions (20), evaluating perceptions of new approaches is a necessary precursor to testing efficacy (e.g., randomized control trials). Stakeholder feedback not only identifies areas of strength but also identifies potential challenges, enabling iterative refinement to optimize the intervention for real-world settings. Early buy-in from both youth and their support systems enhances the likelihood of sustained use, which is vital for ensuring the success of any intervention. Given the limitations of current treatments for CD, often characterized by low engagement and high dropout rates, gathering stakeholder perceptions represents a critical step toward developing interventions that are impactful and engaging for youth, and feasible for those who implement the intervention (i.e., mental health professionals). This foundational work lays the groundwork for future evaluations, ensuring that interventions are not only scientifically rigorous but also practically relevant and applicable in real-world settings. This is important in the development of technology-based mental health care, such as virtual reality (VR), which offers unique and limitless opportunities to create engaging and ecologically valid interventions (33). Given the challenges of engaging youth with CD in conventional treatments, VR offers a unique opportunity to design engaging and effective interventions, particularly when informed by stakeholders.

## 2 Present study

The present study aimed to evaluate perceptions of Impact VR among key stakeholder groups, including mental health professionals, teachers, caregivers of youth with CD, and youth with CD. Specifically, we assessed self-reported ratings of acceptability, appropriateness, and feasibility following participation in Impact VR to determine its potential as a low-resource, scalable alternative treatment. We hypothesized that Impact VR would achieve an approval threshold of >90% across all measures and participant groups, indicating broad support for its implementation. In addition, we explored correlations between youth demographics, CU traits, and CD symptoms with acceptability, appropriateness, and feasibility ratings to identify factors that might influence perceptions of Impact VR. While we did not formulate specific hypotheses regarding these associations, this exploratory analysis provides insights into how individual characteristics, such as CU traits and CD symptoms, may shape perceptions of the intervention.

## 3 Materials and methods

### 3.1 Participants

A total of 60 participants were recruited across four stakeholder groups: 20 youth diagnosed with CD, 20 caregivers of youth with CD, 20 educators, and 20 child and adolescent mental health professionals. Youth participants (aged 10-17 years) were recruited from a large healthcare network in Virginia and were required to have a current CD diagnosis made by a licensed psychiatrist or psychologist. The majority (60%) of the youth were actively receiving mental health treatment (i.e., outpatient services, counseling, family social worker), and all had received treatment within the past year. Caregivers, primarily mothers (95%), ranged in age from 35 to 56 years (*M_age_* = 41.70) and were eligible to participate if they were English-speaking and over the age of 18. Educators were recruited from mainstream and alternative schools in Virginia, teaching grades 5-12. They were identified through existing research collaborations, and participation was voluntary. Teachers ranged in age from 28 to 59 years (*M_age_* = 43) and were evenly split between male and female participants. Mental health professionals (e.g., school counselors, psychologists) were similarly recruited through community-based organizations and schools, with participants ranging in age from 34 to 55 years (*M_age_* = 41.25), the majority of whom were female (60%). Neither educators nor mental health professionals had prior associations with the youth participants. See **Table 1** for demographics.

**Table 1:** Descriptive statistics.

	Mental Health Professionals (*n*=20)	Teachers (*n*=20)	Caregivers (*n*=20)	Youth (*n*=20)
Mean age (*SD*)	41.25 (7.06)	43.00 (9.57)	41.70 (5.67)	14.10 (2.25)
Gender				
Male	8	10	1	10
Female	12	10	19	10
Ethnicity				
White	11	12	10	10
Black	6	7	6	6
Other	3	1	4	4
Mean years in profession	14.40 (6.20)	14.20 (8.77)	N/A	N/A

Other = Hispanic or Asian.

### 3.2 Procedure

Recruitment occurred through email or phone contact with eligible youth-caregiver dyads, educators, and mental health professionals. Interested participants were provided study details and invited to complete Impact VR and accompanying assessments. Youth participants completed Impact VR in a dedicated laboratory setting under the supervision of a clinical research coordinator, while caregivers completed surveys separately in an adjacent space. Educators and mental health professionals engaged with Impact VR at their schools or clinics and subsequently completed the survey questionnaires. All participants followed the same sequence: consent/assent, engagement with Impact VR, and self-report assessments. Impact VR was delivered on a Meta Quest 2 headset. No participants reported physical usability issues, motion sickness, or discomfort. Prior to participation, all individuals provided informed consent or assent, and the study received approval from the [Blinded for Review] Institutional Review Board. Participants were informed that the goal of the project was to evaluate perceptions of Impact VR and to collect feedback to improve Impact VR as a treatment program for youth with conduct problems. To compensate for their time, each participant received $40 upon completion of the assessments.

### 3.3 Intervention

#### 3.3.1 Impact VR

Impact VR is a cutting-edge, immersive psychoeducational platform designed to improve emotion regulation, emotion recognition, and prosocial behaviors in youth. Leveraging the immersive capabilities of VR, Impact VR uses controlled environments where users can engage in targeted scenarios to develop critical social skills. The program integrates evidence-based psychological principles, such as cognitive-behavioral and dialectical behavioral techniques, alongside immersive, gamified activities to enhance learning and engagement. Emotion recognition tasks help participants identify and interpret facial expressions, even when visual cues are obscured (e.g., characters wearing sunglasses or masks), while emotion regulation strategies progressively teach participants to identify triggers and manage complex emotions like frustration or disappointment. Consistent with findings by Mancuso et al. (39), VR’s capacity to elicit emotions comparable to real-world experiences enhances ecological validity and increases memory retention, ensuring participants’ emotional responses and learning are highly transferable to real-life contexts. Social problem-solving tasks, embedded within a storyline centered on group interactions, encourage participants to recognize emotional states, explore the causes of emotions, and engage in prosocial behaviors to resolve conflicts. The program provides real-time feedback, prosocial reinforcement, and dynamically tailored difficulty levels to ensure tasks remain appropriately challenging without overwhelming participants, preventing floor and ceiling effects. Designed as a flexible, self-guided intervention, Impact VR can be implemented independently in schools, clinics, and community settings or facilitated with the support of a mental health professional. This adaptability makes it particularly suited to underserved populations, reducing reliance on external facilitators while maintaining high levels of engagement.

#### 3.3.2 Theoretical framework and development

The development of Impact VR is grounded in a rich body of research on the underlying mechanisms contributing to CD and CU traits, particularly deficits in emotion recognition and regulation. CD and CU traits are associated with disruptions in emotional processing, including impaired recognition of emotions in others (29, 52, 53). These deficits are often attributed to structural and functional abnormalities in neural circuits governing emotion, such as reduced activity in the amygdala and weaker connectivity with the ventromedial prefrontal cortex (29, 54). These impairments hinder the development of empathy and prosocial behavior, exacerbating antisocial tendencies and contributing to persistent behavioral challenges (55). Emotion recognition deficits in youth with CD and CU traits may also stem from altered attentional processing, as these youth tend to show reduced focus on the eye region of faces (56), which is a key area for interpreting emotional expressions (57). This failure to process emotional cues disrupts social learning and fosters insensitivity to others’ emotions, which are critical for adaptive social interactions. Studies further suggest that deficits in recognizing fear may diminish the social feedback necessary for developing guilt and remorse, perpetuating antisocial behaviors over time (58). Further, youth with CU traits have been found to misinterpret neutral or ambiguous facial expressions as hostile (59), which is found to increase the risk of violent behavior (60).

In addition to recognition impairments, youth with CD and CU traits also exhibit profound difficulties in regulating their emotional responses (19), particularly in high-stress or conflict-laden scenarios (61). These emotion regulation deficits often manifest as heightened impulsivity, poor frustration tolerance, and difficulty modulating aggressive responses. Together, deficits in emotion regulation and emotion recognition hinder the ability to navigate social situations and maintain prosocial behavior adaptively.

Given this evidence, Impact VR prioritizes emotion recognition and regulation as foundational components of its intervention framework. By targeting shared neurocognitive processes underlying CD and CU traits, the program aims to address deficits that span multiple disorders, including disruptive behavior problems and neurodevelopmental disorders, all of which have deficits in recognizing and regulating emotions (27). This transdiagnostic approach is particularly important given the high rates of comorbidity and the overlapping emotional and behavioral challenges faced by youth with CD.

#### 3.3.3 Impact VR session structure

Impact VR includes four consecutive 20-minute modules, each building on the previous one through a scaffolding approach. The modules address the following objectives:

##### 3.3.3.1 Session 1: building the foundations of emotional understanding

Youth are taught the four primary emotions (happiness, sadness, anger, and fear) as well as neutral expressions, providing a foundational understanding of emotional awareness. Participants are guided by a virtual companion who explains how to identify emotional expressions using specific facial features such as the eyes and mouth. Through interactive tasks, participants practice recognizing emotions in static facial expressions and receive immediate feedback to reinforce their understanding. Mirroring exercises, where participants replicate the expressions they see, help to build self-awareness and empathy. A scenario-based activity further supports learning by presenting social situations where participants identify characters’ emotional expressions and feelings, and explore the causes behind those feelings.

##### 3.3.3.2 Session 2: enhancing emotional awareness and adaptability

This session builds on foundational skills learned in session one by introducing dynamic emotional expressions and increasing the complexity of emotion recognition tasks. Youth learn to recognize emotions even when visual cues are partially obscured, such as when characters wear sunglasses or masks, encouraging them to rely on contextual and partial information. Emotion regulation strategies are introduced, helping participants identify emotional triggers and select appropriate responses to manage emotions effectively. The session concludes with a gamified activity, where participants practice identifying emotions under time constraints and in progressively challenging scenarios, reinforcing both emotion recognition and regulation skills.

##### 3.3.3.3 Session 3: cultivating emotional connections in social settings

This session focuses on the application of emotion recognition and regulation skills in realistic social interactions, emphasizing the development of prosocial behaviors. Participants engage with a central storyline where they work alongside characters to prepare for a birthday party. The activity involves interpreting emotional expressions, identifying the causes of emotions, and engaging in actions to resolve conflicts and improve group dynamics. Role-playing tasks guide participants in understanding how their responses can influence others’ emotional states, fostering empathetic and supportive interactions. Additionally, participants practice identifying and responding to subtle emotional cues, such as changes in tone or body language, within the context of complex social scenarios. These activities are designed to help participants connect their understanding of emotions to real-world social behaviors, building skills that enhance their interpersonal relationships.

##### 3.3.3.4 Session 4: applying emotional mastery in complex situations

In the final session, participants integrate the skills they have developed, applying emotion recognition, regulation, and prosocial behaviors in challenging and dynamic contexts. Advanced emotion regulation strategies are explored, equipping participants to manage their own emotions during high-stakes interactions such as frustration or disappointment. Multi-character social scenarios encourage participants to analyze overlapping emotional states and respond appropriately to resolve group conflicts. The session culminates in a cumulative gamified task that combines nuanced emotional recognition, real-time decision-making, and empathetic responses. This final activity reinforces the skills learned throughout the program and prepares participants to generalize their abilities to real-world interactions.

#### 3.3.4 Session structure for Impact VR

**Figure 1:**
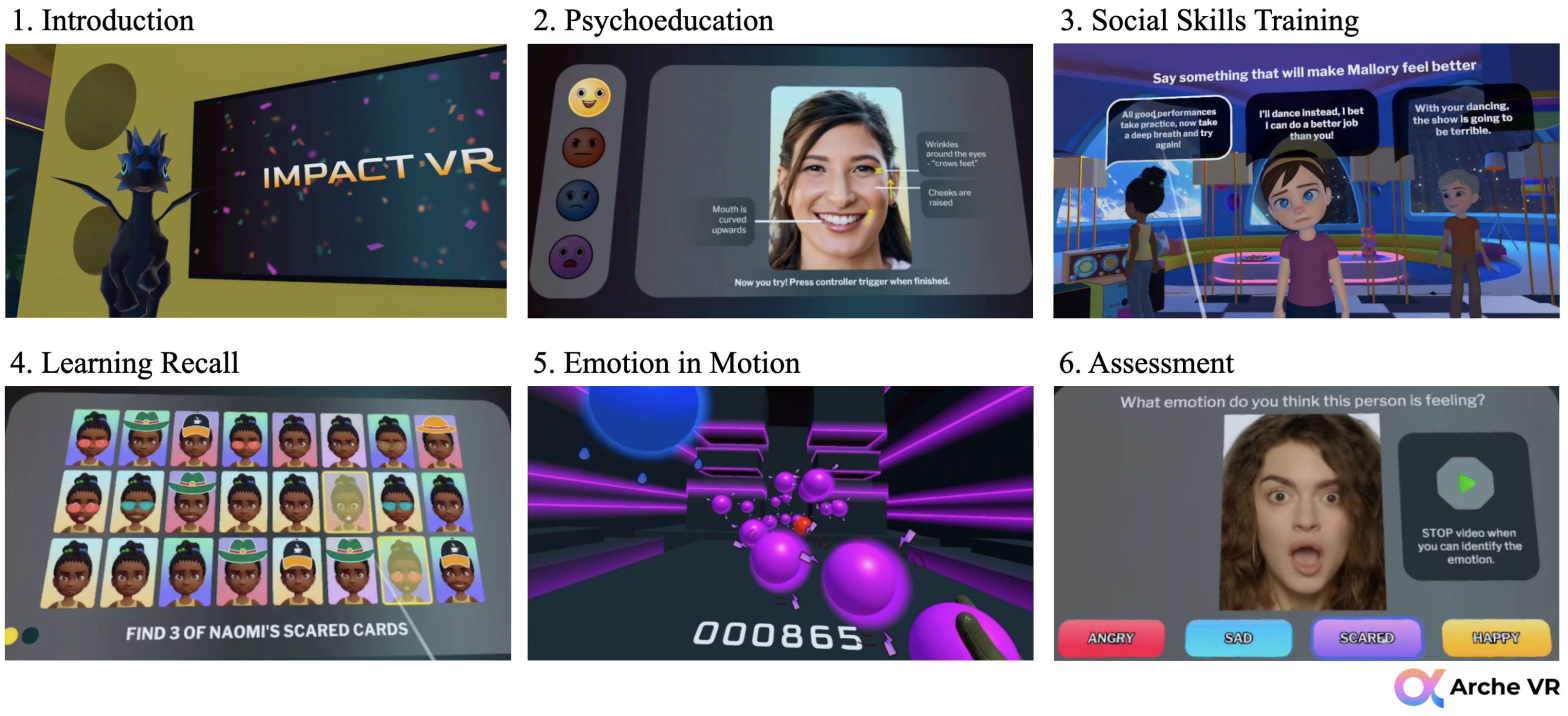
Session module workflow for Impact VR.

Each of the four sessions follows the same modular outline (see **Figure 1**).

##### 3.3.4.1 Introduction

Each session begins with guidance from a virtual companion character, who introduces the session’s focus and learning objectives. This segment sets the stage for the activities by reviewing key concepts from previous sessions and highlighting the specific skills participants will develop. The companion ensures participants are oriented to the session’s tasks and provides context for their importance.

##### 3.3.4.2 Interactive psychoeducation

Youth engage in structured activities that focus on practicing emotion recognition and regulation. These tasks involve identifying and interpreting emotional expressions in both static and dynamic contexts, such as recognizing facial cues (e.g., eyes, mouth) and understanding subtle changes in expressions. Immediate feedback is provided to reinforce learning and encourage skill mastery.

##### 3.3.4.3 Social skills training

Participants immerse themselves in a storyline-based scenario, such as helping characters prepare for a birthday party. Within this context, participants identify the emotions of virtual characters, analyze the causes of these emotions, and apply prosocial behaviors to resolve interpersonal conflicts. This activity emphasizes real-world application by illustrating how emotion recognition and regulation skills can improve social dynamics.

##### 3.3.4.4 Learning recall

To solidify the session’s key concepts, participants complete a recall-based game to reinforce the learned material. For example, they may identify emotional faces in a timed challenge, integrating lessons on static and dynamic emotion recognition while managing distractions.

##### 3.3.4.5 Emotion in motion game

Each session concludes with a dynamic and motivational game that integrates learning, fun, and physical activity. Participants aim to catch as many flying balls as possible, synchronized with an upbeat song about emotions and emotional experiences. The balls change colors to represent emotions in the song (e.g., blue for sadness, red for anger), and participants gain points for catching the correct color while losing points for incorrect choices. This interactive activity reinforces key emotional concepts. Scores are tracked across sessions, challenging participants to improve their performance and fostering a sense of excitement and motivation.

##### 3.3.4.6 Assessment (optional)

Users may finish Impact VR with a brief assessment of emotion recognition using static and dynamic facial expressions of youth and adults.

### 3.4 Measures

#### 3.4.1 Acceptability of intervention measure

The AIM is a four-item measure that assesses the acceptability of the intervention and is written at a 5^th^-grade reading level (62). The AIM was developed based on Proctor et al.’s (63) description of acceptability, given that a treatment, service, practice, or innovation is agreeable, palatable, or satisfactory. Acceptability is critical in promoting adherence to interventions and avoiding high rates of attrition, particularly when delivered in healthcare settings (64). The scale uses a 5-point Likert scale ranging from “Completely Disagree” (1) to “Completely Agree” (5). Items included: “Impact VR meets my approval”, “Impact VR is appealing to me”, “I like Impact VR”, and “I welcome Impact VR”. Higher scores indicate greater levels of acceptability. In addition to raw and total scores, we calculated the percentage approval rating from a binary score of 1 = approved (scores of 4 “Agree” and 5 “Strongly Agree”) and 0 = not approved (scores 3 “Neither Agree or Disagree”, 2 “Disagree”, or 1 “Strongly Disagree”). The approval rating was calculated by multiplying the number of acceptable scores by 25. The threshold for approval was considered met with an approval rating of 90% or higher (64). The total AIM score has reported good reliability in past research (0.85; 62) and in the present study (Cronbach’s α = 0.83).

Youth participants also completed an additional acceptability measure based on Sekhon et al.’s (64) theoretical framework of acceptability for healthcare interventions. This included targeted yes/no questions related to (1) burden (e.g., “Did you experience any negative physical symptoms?”), (2) cultural relevance (e.g., “Is Impact VR culturally sensitive and informed?”), (3) intervention coherence (e.g., “Were the instructions clear and understandable?”), and (4) opportunity cost (e.g., “Do you feel Impact VR is a good use of your time?”). Lastly, participants were asked about the skills they learned and their perception of how these skills could improve their relationships.

#### 3.4.2 Intervention appropriateness measure

The IAM is a four-item survey based on Proctor et al.’s (63) definition of intervention appropriateness, which is the perceived fit, relevance, or compatibility of the innovation or evidence-based practice for a given practice setting, provider, or consumer; and/or perceived fit of the innovation to address a particular issue or problem. Items included: “Impact VR seems fitting”, “Impact VR seems suitable”, “Impact VR seems applicable”, and “Impact VR seems like a good match”. Participants were provided an overview of Impact VR and its intended population (youth 10-17 with CD) before completing the intervention. A 5-point Likert scale ranging from “Completely Disagree” (1) to “Completely Agree” (5) was used, with higher scores indicating greater levels of appropriateness. The percentage approval rating was calculated from a binary score of 1 = Approved (scores higher than 4 “Agree”) and 0 = Not Approved (scores 3 “Neither Agree or Disagree” or less). A >90% approval rating was considered the threshold for meeting approval. The IAM has demonstrated strong reliability (Cronbach’s α = 0.94) in the current study.

#### 3.4.3 Feasibility of intervention measure

The FIM is a four-item survey that assesses the extent to which a new treatment can be successfully used or carried out within a given agency or setting (62). Caregivers were asked to report on the feasibility of the use of Impact VR within their homes, and youth were asked to report the feasibility in their homes and schools. Teachers were asked to report on the feasibility of using Impact VR within the school environment. Mental health providers were asked to report on the feasibility of the use of Impact VR within the clinic/school counseling environment. The four items included “Impact VR is easily implementable”, “Impact VR is resource efficient”, “Impact VR is deployable”, and “Impact VR is easy to use”. The FIM uses a 5-point Likert ranging from “Completely Disagree” (1) to “Completely Agree” (5), with higher scores indicating greater appropriateness. Approval scores were calculated using the same method as the AIM and the IAM, with the same 90% threshold for approval. Prior research has found the FIM has good reliability (Cronbach’s α = 0.88; 62), which was also found in the present study (Cronbach’s α = 0.84).

### 3.5 Callous-unemotional traits

The Inventory of Callous-Unemotional Traits (65) is a 24-item self-report assessment that uses a four-point Likert scale from 0 (“Not At All True”) to 3 (“Definitely True”) to assess CU traits. In the present study, the ICU total score yielded good internal consistency (Cronbach’s α = 0.86), similar to prior studies (61, 66). The ICU mean score and dispersion (*M* = 29.10; *SD* = 9.7) were similar to prior studies, including those from a residential facility program (*M* = 25.74, *SD* = 7.95; 67).

### 3.6 Conduct disorder symptoms

The conduct disorder facet of the Proposed Specifiers for Conduct Disorder (PSCD; 68) was used to assess CD symptoms. The CD scale includes five items, which are rated on a 3-point Likert scale (0 = “Not True”, 1 = “Sometimes True”, and 2 = “True”). Prior research has found the scale to have good reliability (69), as found in the present study (Cronbach’s α = 0.84).

### 3.7 Data analytic plan

First, we explored descriptive statistics for youth acceptability across the four dimensions (burden, cultural relevance, intervention coherence, opportunity cost) and anticipated self-improvement. Next, we explored descriptive data across groups (mental health professionals, teachers, caregivers, and youth) on the acceptability (AIM), appropriateness (IAM), and feasibility (FIM) scales. Using the AIM, IAM, and FIM, we tested the approval rating of acceptability, appropriateness, and feasibility using a cut-off score of 90%. To further our understanding of youths’ perception of the intervention and if these perceptions were driven by demographics or CU traits and CD symptoms, we explored the correlations between demographics (age, gender, and ethnicity), CU traits, and CD symptoms with scores on the AIM, IAM, and FIM. For continuous variables, Pearson’s correlation coefficients were calculated, while Spearman’s rho was employed for associations involving dichotomous variables. There were no missing data on these scales. *A priori* power analysis, conducted in R (70) using the pwr package (71), determined that with a sample size of 20, the study is sufficiently powered to detect correlations of moderate-to-large effect sizes (*r*≥0.58) at α = 0.05 and power = 0.80.

## 4 Results

### 4.1 Youth-report of 4-domains of acceptability and anticipated self-improvement effects

**Table 2** displays the descriptives for youths’ perceptions of acceptability across the four domains and anticipated self-improvement effects. All youth reported that Impact VR was culturally sensitive and relevant. Youth (100%) reported not experiencing any burden in response to Impact VR, and 90% of youth felt that the intervention was coherent, with two participants (10%) reporting that the instructions were unclear. Participants reported favorably of the opportunity cost items, with 100% of youth reporting they felt Impact VR was a good use of their time and would rather complete Impact VR than traditional mental health care services, including counseling, therapy, and case management. Most youth (95%) reported learning new skills during Impact VR. Overall, youth positively reported that the skills learned during Impact VR would improve their relationship with their friends (100%), parents (90%), and teachers (90%). Lastly, all youth reported feeling more confident that they could recognize emotions in others (see **Table 2** for items).

**Table 2:** Frequencies of acceptability ratings.

Cultural Relevance	Yes	No
Is the content culturally sensitive and relevant?	100%	0%
Does the content avoid stereotypes and promote positive, accurate portrayals of all cultural groups?	100%	0%

Burden	Yes	No
Did you experience any negative physical symptoms?	0%	100%
Was the experience too long?	0%	100%

Intervention Coherence	Yes	No
Is the intervention easy to follow and understandable?	90%	10%
Were the instruction clear and understandable?	90%	10%

Opportunity Costs	Yes	No
Do you feel Impact VR is a good use of your time?	100%	0%
Would you rather complete Impact VR instead of traditional mental health services (e.g., counseling, therapy, case management)?	100%	0%

Anticipated Self-Improvement Effects	Yes	No
During Impact VR, were you able to learn new skills?	95%	5%
Do you think the skills taught in Impact VR can improve your mental health?	90%	10%
Do you think the skills taught in Impact VR can improve your relationships with your friends?	100%	0%
Do you think the skills taught in Impact VR can improve your relationships with your teachers?	95%	5%
Do you think the skills taught in Impact VR can improve your relationships with your parent(s)?	90%	10%
Do you feel more confident that you can recognize emotions in others?	100%	0%

### 4.2 Acceptability, appropriateness, and feasibility of Impact VR

**Table 3** displays the descriptives for the AIM, IAM, and FIM. Across each item of the AIM, mental health professionals (*n*=20), teachers (*n*=20), caregivers (*n*=20), and youth with CD (*n*=20) reported average scores in the “Agree” category, resulting in a high approval rating (95.00% - 100%) for the acceptability of Impact VR. Results were consistent for IAM and FIM, with scores across groups averaging in the “Agree” category with a high approval rating for intervention appropriateness (98.75% - 100%) and feasibility of the intervention (97.50% - 100%). Across all the AIM, IAM, and FIM scales, none (0%) of the participants reported disagree or strongly disagree.

**Table 3:** Acceptability of intervention measure scores.

Acceptability of Intervention Measure (AIM):	Mental Health Professionals *M (SD)*	Teachers *M (SD)*	Caregivers *M (SD)*	Youth *M (SD)*
1. Impact VR meets my approval	4.75 (0.44)	4.75 (0.44)	4.55 (0.61)	4.40 (0.59)
2. Impact VR is appealing to me	4.75 (0.44)	4.65 (0.49)	4.25 (0.55)	4.65 (0.49)
3. I like Impact VR	4.60 (0.50)	4.75 (0.44)	4.50 (0.51)	4.55 (0.60)
4. I welcome Impact VR	4.95 (0.22)	4.85 (0.37)	4.50 (0.51)	4.45 (0.69)
AIM Total Score	19.05 (0.76)	19.00 (0.86)	17.75 (1.07)	18.05 (1.47)
AIM Approval Percentage	100%	100%	97.50%	95.00%
Intervention Appropriateness Measure (IAM)				
1. Impact VR is fitting	4.70 (0.44)	4.60 (0.50)	4.20 (0.41)	4.55 (0.61)
2. Impact VR is suitable	4.60 (0.50)	4.50 (0.51)	4.25 (0.55)	4.55 (0.51)
3. Impact VR is applicable	4.50 (0.51)	4.50 (0.51)	4.25 (0.44)	4.55 (0.51)
4. Impact VR is a good match for the target population	4.60 (0.50)	4.75 (0.44)	4.25 (0.44)	4.50 (0.52)
IAM Total Score	18.40 (1.39)	18.35 (1.03)	16.95 (0.88)	18.15 (1.39)
IAM Approval Percentage	100%	100%	98.75%	98.75%
Feasibility of Intervention Measure (FIM)				
1. Impact VR is easily implementable	4.75 (0.44)	4.40 (0.60)	4.25 (0.55)	4.55 (0.51)
2. Impact VR is resource efficient	4.30 (0.47)	4.70 (0.47)	4.20 (0.52)	4.45 (0.51)
3. Impact VR is deployable	4.70 (0.57)	4.50 (0.69)	4.30 (0.47)	4.35 (0.49)
4. Impact VR is easy to use	4.55 (0.60)	4.15 (0.68)	4.45 (0.51)	4.55 (0.51)
AIM Total Score	18.30 (1.45)	17.75 (1.45)	17.20 (1.23)	17.90 (1.02)
Acceptability Percentage	97.50%	98.75%	97.50%	100.00%

Item scores range from 1-5.

### 4.3 Correlations among scores, demographics, and CD and CU traits for youth

Correlations displayed in **Table 4** show that age was negatively related to the AIM total scores but not significantly related to the IAM or the FIM scores. Both gender and minority ethnicity were unrelated to total scores on the AIM, IAM, or the FIM. CU traits were positively related to the AIM and FIM total scores. CD was positively related to the AIM but unrelated to the IAM and FIM. In summary, younger youth, and those with higher CU traits and CD reported greater acceptability of Impact VR. Youth higher on CU traits also reported greater levels of feasibility. Importantly, the approval of Impact VR as an acceptable, appropriate, and feasible intervention is not related to minority ethnicity or gender.

**Table 4:** Correlation among main study variables and demographics.

	AIM	IAM	FIM
Age	-0.69***	-0.02	-0.11
Gender^†^	0.03	0.13	0.28
Ethnicity^†^	0.08	-0.08	0.14
CD	0.66**	0.31	0.34
CU traits	0.60**	0.34	0.52*

Ethnicity (1= White; 0 = Minority ethnicity); Gender (1= Male; 0 = Female); CD = PSCD conduct disorder scale; CU traits = ICU total score. ^†^Spearman’s rho; *p<.05; **p<.01; ***p<.001.

## 5 Discussion

The goal of the present study was to understand if Impact VR was an acceptable, appropriate, and feasible intervention for youth with CD across key stakeholder groups. Results from 20 child-parent dyads, 20 educators, and 20 child and adolescent mental health professionals (e.g., school counselors and psychologists) supported the approval threshold for acceptability, appropriateness, and feasibility for Impact VR. Although we did not form expectations on correlations among study variables, we found greater acceptability among younger youth with CD. Interestingly, higher levels of CU traits and CD were positively correlated with greater acceptability, suggesting that Impact VR may be of particular interest to high-risk youth. Across stakeholder groups, Impact VR was found to be culturally sensitive, and among youth it was the preferred treatment option for mental health care when compared to traditional methods.

### 5.1 Implications

Stakeholder engagement is a critical step in the development of interventions, as it ensures that the intervention is practical and relevant in real-world settings. A user-centered approach increases the likelihood that behavior change interventions are user-relevant, thoughtfully designed, readily implementable, and ultimately more effective (46). Assessing factors such as acceptability, appropriateness, and feasibility improves the understanding of whether an intervention resonates with key stakeholders, including youth, caregivers, and practitioners. If an intervention is difficult to implement, poorly received, or does not meet the needs of its intended users, its potential impact is significantly diminished, regardless of its demonstrated effectiveness (46). For example, stakeholder feedback has been instrumental in tailoring interventions to diverse populations, ensuring that cultural and contextual factors are addressed (72). By evaluating these factors early, developers can identify potential barriers to adoption and refine the intervention to enhance its scalability and sustainability. This proactive approach is particularly important for novel methods like VR-based interventions, which must demonstrate not only their efficacy but also their practical utility and user appeal. Ultimately, interventions that align with stakeholder priorities and operational realities are more likely to be adopted, ensuring their broader impact and reach. Incorporating stakeholder feedback bridges the gap between research and practice by ensuring that the development of interventions aligns with the needs and preferences of the intended users, fostering greater engagement and practical relevance.

Although preliminary, these results demonstrate Impact VR as a low-barrier, innovative, and novel approach for this target population. The high scores among each stakeholder category point to the feasibility, appropriateness, and acceptability of Impact VR for use in a wide range of facilities, including inpatient and outpatient mental health clinics, hospitals, mainstream and alternative schools, and in the home. The finding that younger participants reported higher levels of acceptability suggests that this age group may be more engaged with the immersive and gamified nature of Impact VR. Similarly, we found greater acceptability among youth with high CU traits and CD symptoms, suggesting that this modality may meet the interests of youth high on these traits. Greater engagement could promote treatment adherence by encouraging continued participation across multiple sessions, a critical factor for intervention success. This aligns with prior research indicating that user acceptability is a key predictor of adherence interventions (see 64). These implications suggest that VR may be an effective modality for promoting greater adherence to treatment interventions among CD youth. The present study demonstrated that youth overwhelmingly reported a preference for Impact VR (100%) for their mental health treatment compared to traditional therapies. These findings build on prior research demonstrating a preference for VR tasks among youth with attentional and emotional deficits (45). Given that youth with CD and CU traits are often perceived as difficult to engage in treatment (20), these results are encouraging.

The findings that youth with CD reported the intervention as culturally sensitive, relevant, and good use of their time underscore the potential of Impact VR to resonate deeply with this hard-to-reach population. The unanimous perception of Impact VR as culturally sensitive and free of stereotypes underscores its potential for wide applicability and acceptance across diverse populations. This aspect is critical for ensuring the relevance and effectiveness of the intervention in real-world, multicultural contexts. These positive perceptions are critical as they suggest that the intervention not only meets the cultural and experiential needs of the youth but also engages them in a meaningful way, which is often difficult to achieve with traditional therapeutic approaches. The confidence expressed by the youth in the *perceived* skills acquired, particularly in recognizing emotions and improving interpersonal relationships with peers, teachers, and parents, further highlights the potential for Impact VR. This suggests that the immersive and interactive nature of VR promotes positive perceptions of learning and skill acquisition. Consequently, these findings support the continued development and deployment of culturally tailored, VR-based interventions as a viable and innovative approach to treating CD.

There are broader implications of these findings that underscore the advantages of Impact VR as a new modality to promote emotion recognition, regulation, and prosocial behaviors in youth. First, Impact VR is self-guided, customized to adapt to task difficulty, and can be self-administered. The portability and usability of this intervention on affordable VR hardware (versus the use of more costly hardware) make this an ideal intervention to administer at scale in various settings and locations (e.g., inpatient and outpatient clinics, school and home environments, etc.). Second, high acceptability, appropriateness, and feasibility scores across stakeholder groups suggest the potential for widespread deployment to youth in both clinical and non-clinical populations. Although research on mental health interventions in VR, particularly regarding emotion recognition, is in its infancy, these preliminary findings build on prior research demonstrating the potential for enhanced treatment efforts through gamification (43). This study adds to the growing evidence supporting the use of VR-based interventions for youth with CU traits. Impact VR is designed to function both as a standalone strategy and as a complementary tool alongside existing treatments. When used in conjunction with traditional methods, VR-based therapies have the potential to enhance engagement, adherence, and overall effectiveness. For example, in Heikkilä et al.’s (40) case study, VR augmented their existing intervention (CFT; also called PSYCHOPATHY.COMP) by improving emotional insight through immersive exposure, demonstrating how VR can enrich therapeutic outcomes.

Youth with CD often face treatment barriers (73). Existing literature highlights how language processing difficulties and reduced sensitivity to social and emotional rewards contribute to low engagement and high dropout rates in verbally intensive therapies (74). Additionally, other critical barriers, such as stigma associated with mental health treatment (75, 76), limited accessibility to mental health treatment (77), cultural and language barriers (72), and economic barriers (78) may further hinder the success of traditional treatment approaches. VR-based therapies can address these barriers by increasing accessibility, as they can be deployed readily at a low cost and operate without requiring specialized IT infrastructure or mental health networks, making them viable even in resource-limited settings. This approach eliminates geographic constraints and reduces costs associated with traditional in-person therapy sessions (33). A VR approach also engages youth through interactive and immersive gamified elements, which make therapy more appealing and maintain attention in ways that traditional methods may not. Additionally, VR platforms can be tailored to reflect diverse cultural backgrounds and languages, fostering relevant and culturally appropriate environments for participants that reduce stigma and encourage engagement (33). Importantly, evidence-based VR mental health programs ensure consistent implementation regardless of who delivers the treatment by embedding standardized therapeutic protocols, promoting accessible mental healthcare across diverse populations (33).

If Impact VR or other self-guided VR-based mental health interventions prove to be effective, they are likely to offer a scalable and cost-effective alternative to traditional treatments for CD and CU traits. One of the key challenges associated with existing treatments is the substantial cost and the extensive resources required for implementation. These interventions can cost between $5,000 and $10,000 per youth annually (25), requiring intensive, year-round support from trained professionals. In contrast, interventions targeting the mechanisms of disorders, like Impact VR, leverage self-guided technology, reducing the need for ongoing professional oversight and minimizing logistical barriers. If Impact VR is effective, the costs of distributing and maintaining a VR-based intervention will be significantly lower, largely because it does not require mental health professionals to administer it. However, future research should explore a cost-benefit analysis of VR interventions, including the initial expenses of VR hardware (i.e., headsets), subscription, and potential staff-related costs (i.e., training). By addressing these cost and resource barriers, Impact VR holds the potential to make evidence-based mental health care more accessible, particularly in under-resourced schools and communities.

Naturally, there are several limitations to consider. First, this study was not intended to evaluate the effectiveness of Impact VR on outcomes; therefore, although youth perceived themselves as having improved, this needs to be rigorously tested. Future research should incorporate objective measures, such as multi-informant assessments or performance-based tasks, to provide a more comprehensive evaluation of the intervention’s impact. Additionally, randomized controlled trials with active control conditions are needed to disentangle the specific effects of Impact VR from potential placebo effects or general engagement-related improvements. Thus, future research using robust methodological research designs (e.g., randomized control trials) is needed to evaluate its effectiveness in reducing CD and CU traits. Second, while the present study aimed to understand perceptions of Impact VR from key stakeholder groups, only quantitative data was sourced for the present study. Additional exploration into the acceptability, appropriateness, and feasibility of this intervention would benefit from a mixed methods design. Third, although this intervention is geared towards youth with CD, administering this intervention to youth with similar-type emotion recognition difficulties (e.g., autism, etc.) may be beneficial to understand its widespread applicability and suitability to a wide range of youth who present with emotional deficits. In addition, while this paper focused on descriptive statistics of perceptions of Impact VR, the exploratory correlational analyses among the main study variables were only powered to detect large effect sizes. As a result, smaller but potentially meaningful associations may not have been identified. A key limitation of the present study is the lack of objective measures to validate the self-reported perceptions of Impact VR’s effectiveness. While the study relied on participant-reported outcomes to assess acceptability, feasibility, and appropriateness, these measures may be influenced by social desirability bias. For instance, participants may have provided positive ratings on mental health and relationships due to the novelty of the VR intervention rather than its actual efficacy (79). The immersive and engaging nature of VR might have created an expectation of improvement, which could have biased participants’ responses. However, it is also possible that the immersive nature of VR increased skill retention, as found in prior VR research (e.g., 80), hence the high ratings of perceived improvements among youth. Nevertheless, the present study aimed to understand perceptions of Impact VR from key stakeholder groups, and overall, these groups reported the intervention as acceptable, appropriate, and feasible.

## 6 Conclusion

Impact VR shows promise as an engaging intervention for youth with CD and CU traits. The study demonstrates broad support across the mental health and education continuum, with positive endorsements from teachers, mental health professionals, caregivers, and youth with CD. Notably, youth participants reported that they could see the benefits of Impact VR in improving their mental health and relationships with others, highlighting the program’s *perceived* effectiveness in addressing key areas of social and emotional development. The strong support from key stakeholders highlights the potential of Impact VR to be deployed successfully in diverse settings (e.g., homes, schools, and clinics), positioning Impact VR as a promising tool for addressing the multifaceted challenges associated with CD in an impactful manner.

## Data Availability

The datasets presented in this article are not readily available because the data presented here are not publicly accessible due to ethical and privacy concerns. Data may be made available upon contacting the first author and under the conditions of a Data Use Agreement and pre-approval from an Institutional Review Board. Requests to access the datasets should be directed to Nicholas.thomson@vcuhealth.org.
